# PATL: A RFID Tag Localization based on Phased Array Antenna

**DOI:** 10.1038/srep44183

**Published:** 2017-03-15

**Authors:** Lanxin Qiu, Xiaoxuan Liang, Zhangqin Huang

**Affiliations:** 1 Beijing Advanced Innovation Center for Future Internet Technology, Beijing University of Technology, Beijing 100124, China; 2 Beijing Engineering Research Center For IoT Software and Systems, Beijing University of Technology, Beijing 100124, China

## Abstract

In RFID systems, how to detect the position precisely is an important and challenging research topic. In this paper, we propose a range-free 2D tag localization method based on phased array antenna, called PATL. This method takes advantage of the adjustable radiation angle of the phased array antenna to scan the surveillance region in turns. By using the statistics of the tags’ number in different antenna beam directions, a weighting algorithm is used to calculate the position of the tag. This method can be applied to real-time location of multiple targets without usage of any reference tags or additional readers. Additionally, we present an optimized weighting method based on RSSI to increase the locating accuracy. We use a Commercial Off-the-Shelf (COTS) UHF RFID reader which is integrated with a phased array antenna to evaluate our method. The experiment results from an indoor office environment demonstrate the average distance error of PATL is about 21 cm and the optimized approach achieves an accuracy of 13 cm. This novel 2D localization scheme is a simple, yet promising, solution that is especially applicable to the smart shelf visualized management in storage or retail area.

Radio Frequency Identification (RFID) is one of the indispensable technologies in the Internet of Things^1^ area and has been widely used in storage, logistics and retail fields. In the real applications, RFID system is mainly composed of reader and tag. The tag that stores the commodity information and an unique identification code is installed on the object. Previously deployed reader communicates with tags to read and write the tags’ information by transmitting wireless electronic signal. By doing so, it is capable of identifying, tracing and managing the information of commodity. Comparing to the traditional identification technologies such as bar code or QR (Quick Response) code, RFID has the advantage of efficient batch processing. RFID tags can be either active or passive. Active tags require embedded battery to actively transmit data to the reader, while battery-free passive tag collects power from the read’s transmission wave and reflects its self-information back to the reader. Passive tag can be small enough to integrate into various daily use objects. It has been reported that apparel application alone will demand 4.6 billion RFID labels in 2016^2^. With the rise of the emerging 3D printing technology, Microsoft and Disney Research attempted to directly incorporate RFID-like embedded encoding information into objects during manufacturing process as well^3^,^4^, which may enable the objects to be uniquely identifiable and sensible at the source in the future.

The RF signals that the tags back-scatter not only include the basic commodity information, but also a lot of information about the environment around the objects (e.g. Received Signal Strength Indication (RSSI), Time of arrival (ToA) and Angle of Arrival (AoA) etc.) which can be used to analyse the objects spatial information. If tags can be localized in 2D or even 3D, a completely new and useful application area opens up to provide more intelligent and diverse visualization services to the users^5^. One of the classic scenarios is the visualized smart shelf^6^. A reader being capable of localizing RFID tags and mounted on the ceiling can build an objects visualized map to track the inventory asset with less labor, faster inventory counting and higher visibility of individual items^7^.

Nowadays, there are two methodologies of location awareness for RFID: ranging based and range-free based. Ranging method usually requires the deployment of multiple RFID readers in the environment, and using the geometric relationship of the distance (or angle) to solve nonlinear equations and calculate the target location^8^,^9^,^10^. The traditional fingerprinting based range-free methods^11^,^12^,^13^ require a large number of reference tags with known locations as anchor nodes in the environment to estimate the location of the target tag. Considering to the complex multipath interference problem in the environment, the locating precision of ranging method is usually lower than range-free method. However, due to the high deployment consumption, range-free method is not conductive to the practical applications. As a consequence, this paper aims to propose a range-free method without any reference tags to not only reduce system cost, but also remain a high precision level.

On the other hand, comparing to other positioning technology, AoA can provide a higher positioning accuracy and multipath interference capability for UHF RFID^9^. In order to obtain the angle of arrival of the tag, the reader antenna plays an important role in it. The RF electromagnetic field that the communication environment of RFID system depended on requires the antenna to transmit RF energy into the space. However, general RFID system implementing single circular polarized antenna always shows a poor radiation pattern. It also leads to weak direction performance which is unable to judge the exact angle of the tag and provide AoA estimation. As a consequence, previously related works of AoA methods remain in the theoretical stage. For example, the authors of ref. 9 assumed to use four readers that are placed on the vertices of a tetrahedron to give the angle of the tag and the geometric relationship was used to explain 3D positioning of the tag in theory. Manzoor *et al*.^14^ relied on reference tags to give the tag’s angle and further used the weighting method to estimate the label position.

In recent years, some studies try to combine RFID system with the theory of phased array antenna in radar detection field by designing a new phased array antenna which is suitable for UHF frequency band in order to implement the measurement of the angle of arrival in the reality situation. Bantin *et al*.^15^ utilized phase comparison technique and RF tags located on the guideway to control train automatically. The principle is to measure the differential phase between two array elements that are combined in both a sum and a difference configuration. Ref. 16 introduced a linear array antenna composed of 8 monopole antenna units which can scan the environment with specific direction from 45 to 135 degree. Further refs 17 and 18 deployed multiple phased array antennas at different locations in the environment. Each array antenna is capable of providing a specific linear radiation direction. Both of them proposed range-free method to estimate the tags position by measuring multiple angles of arrival of the tag. The distance error between the estimating position to the ground truth is about 21 *cm*.

In this paper, we firstly propose a range-free tag localization method based on two-dimensional phased array antenna named PATL (Phased Array antenna Tag Localization). Being different from ref. 15 which was used for locating one tag in dynamic environment, our method is mainly applied to locate tags in the region where lots of static tags need to be inventoried. Additionally, by comparing to refs 16, 17, 18, our method can locate several tags at the same time since we use two-dimensional phased array antenna. The two-dimensional phased array antenna provides our method with a better scanning scope. Our method takes advantage of the adjustable radiation angle of the phased array antenna to scan the surveillance region in turns. Through the statistics of the number of tags in different antenna direction area, we use a weighting algorithm to calculate the two-dimensional position of the tag. This method can be applied into the real-time location of multiple targets without the usage of any reference tags or additional readers. Additionally, we present an optimized weighting method EPATL (Enhanced PATL) based on RSSI to increase the locating accuracy. We use a commercial UHF RFID reader which is integrated with phased array antenna to derive experimental verification from an indoor office environment. This paper proceeds as follows. In Section 2 we discuss related work. We describe the design of our system model in Section 3. The PATL and EPATL localization scheme is discussed in Section 4 and 5. Section 6 then presents real-world experiments with EPC C1G2 passive tags and a COTS RFID reader. Finally, Section 7 concludes the paper.

## Backgrounds of Phased array antenna

Comparing to other positioning technologies, angle of arrival based localization can provide a higher positioning accuracy and multipath anti-interference capability for UHF RFID^9^.

Phased array antenna is an array of antennas in which the relative phases of the respective signals feeding the antennas are set in such a way that the effective radiation pattern of the array is reinforced in desired directions and suppressed in undesired directions^19^. According to the radiation formation, which can be interpreted as the number of desired directions, phased array antenna can be divided into single beam and multiple beams. It can be also divided into linear phased array antenna, 2D phased array antenna etc, in terms of the distribution of antenna elements. Although different phased array antennas have their own characteristics, the basic principles are the same^17^. In this paper we mainly discuss about the single beam system. Additionally, 2D antenna can be regarded as a combination of multiple linear phased array antennas, so we firstly look into the linear antenna as an example to analyze the principle of phased array antenna.

**Figure 1:**
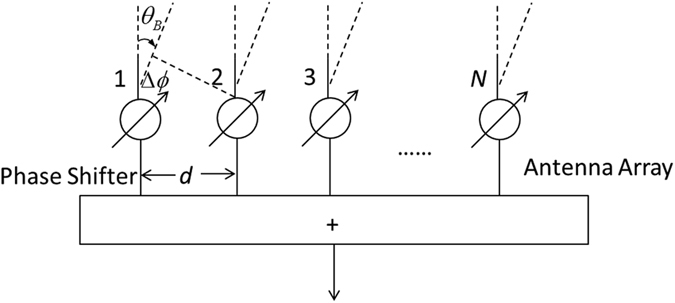
Principle of phased array antenna.

As shown in the Fig. 1, linear antenna elements are usually distributed uniformly in a straight line. They usually have fixed array element number and element interval size. Assuming that the linear antenna array is composed of *N* antenna elements and the tag’s incidence direction relative to the *n*^*th*^ array element is *θ*_*n*_, the signals received by the linear antenna can be expressed as below due to the backscatter communication feature:


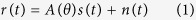


where, *A*(*θ*) is the channel impulse response function. It is a set of all the receive signal components from each antenna element.


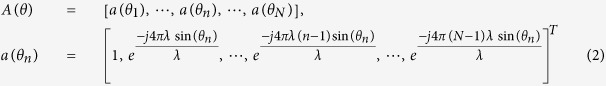


Here *d* and *λ* respectively represent the antenna element interval and the carrier wave length^20^. *e*^−*j*(·)^ describes a complex exponential signals with phase value as (·). It can be seen that the received phase value of each antenna element is determined by the incident angle of the tag and the range difference relative to the first antenna unit. When the antenna element interval *d* and the tag-antenna distance *r* hold the relation of *d* ≪ *r*, the incident angle differences among different antenna elements will be so small that we can unitedly use *θ*_*B*_ to replace the incident angles *θ*_1_, *θ*_2_ … *θ*_*n*_. Then the phase difference between two adjacent elements can be expressed as


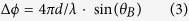


To this end, if we add a phase shifter for each radiating element, we can make the phase difference satisfying the afore-mentioned relationship in formula (3) by shifting the phase of the signal emitted from each elements. Thus it provides us a narrow beam which implements the radiation angle of the antenna array exactly equaling to the tag’s incident angle *θ*_*B*_ = sin^−1^(Δ*ϕ*/4*πd*). At this point, the radiation pattern function of the linear array, which indicates that the radiating amplitude in a certain direction *θ* with beam direction *θ*_*B*_, can be expressed as


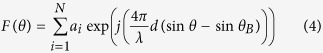


where the excitation current amplitude corresponding to each antenna element is denoted as *a*_*i*_(*i* = 1, …, *n*, …, *N*). If the current amplitude is all the same, we can treat it as *a*_*i*_ = 1/*n* to simplify the function^20^.

The tag’s incident angle *θ*_*B*_ is also defined as AoA, which is treated as an efficient measurement for tag locating^9^. Generally, *θ*_*B*_ is unknown. As described above, phased array antenna can provide constructive/destructive interference so as to steer the radiation beams in the desired direction. Thus by controlling the feeding phase value to transform the beam direction, phased array antenna can scan the entire surveillance region. As soon as the tag is read, the beam direction at this moment is determined as the angle of arrival.

A two-dimensional phased array is useful for the 2D tag localization scenario. The shape of two-dimensional phased array can be rectangular, round, oval or polygon, but its radiation pattern function is regarded as the product of multiple uniform linear arrays. Using two linear arrays in horizontal and vertical direction as an example, the radiation pattern function of the two-dimensional phased array composed by these two linear arrays is |*F*(*θ*, φ)| = |*F*_1_(*θ, φ*)|·|*F*_2_(*θ*)|. |*F*_1_(*θ*, φ)| and |*F*_2_(*θ*)| are used to indicate the radiation pattern function in linear horizontal and vertical direction respectively. By changing the feeding phase of multiple linear arrays simultaneously, we can realize the beam direction scanning on two-dimensional plane. If the 2D phased array is implemented on the ceiling with a height *H* to the ground and we use (*θ*_*B*_*, φ*_*B*_) the beam steering angle as soon as the phased array detecting the tag, the center coordinate of the phased array antenna readable region on the ground can be expressed as below. It can be also treated as the tag’s 2D position.


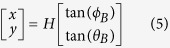


## PATL Positioning Method

### System composition

**Figure 2:**
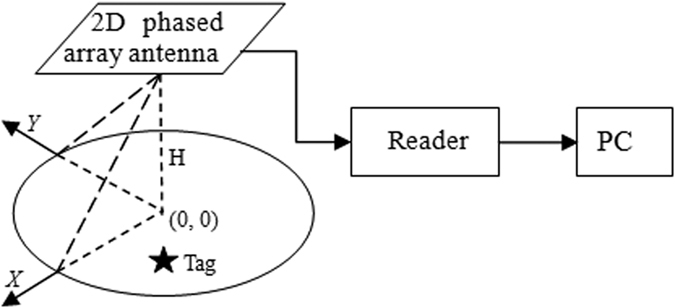
2D phased array antenna RFID positioning system.

As shown in Fig. 2, in this paper, the positioning system is composed of a UHF RFID reader, a two-dimensional phased array antenna, an unknown passive RFID tag which is to be measured, and a computer for data processing and visualization. The phased array antenna is a duplex one, which the reader uses to transmit commands to the tag and receive the tag’s backscatter information at the same time. Backscatter data from different tags can be classified based on the unique EPC code for time-sharing processing. In this paper, we treat the radio frequency antenna and the reader as one entirety, and do not make a distinction between their positions. We establish a coordinate system as shown in the Fig. 2, in which the coordinate origin is the projection of the antenna on the ground. The reader system is suspended in the air to scan the area below it, and the area size is related to the radiation capability of the antenna. The height of the tag and the antenna is set to be known. The result of the 2D coordinates of the tag is relative to the antenna position under this global coordinate system. The final position of the tag will be displayed directly on the PC. In the process of positioning, tags and readers are static.

### PATL

Considering the feature of the 2D phased array antenna where the beam direction is known and adjustable, we propose a range-free weighting method PATL to calculate the two-dimensional coordinates of the tag. We set the known antenna projection coordinate on the ground as (0, 0). Ideally, if the antenna beam can precisely modify its beam direction to scan the entire two-dimensional plane and the radiation lobe is enough narrow, then when the antenna receives the tag’s backscatter signal on a certain angle, we can directly consider that the coordinate of the tag as calculated by formula (5).

**Figure 3:**
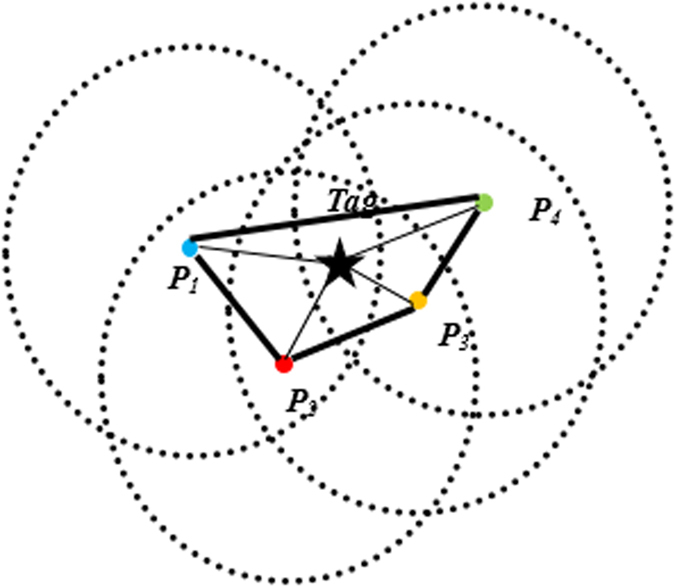
The basic principle of PATL. The tag’s position can be calculated as the centroid of the quadrangle consisting of the line segments among *P*1, *P*2, *P*3 and *P*4, where *P*1, *P*2, *P*3, *P*4 respectively represent the center coordinate on the ground of 4 different antenna beams.

However, according to the radiation function (4), it shows that the lobe of antenna usually has a certain width in reality. As shown in the Fig. 3, we assume the antenna radiation lobe range on the ground is a circle with a radius. When the tag is in this circle, no matter how far the distance is between the tag position and the radiation center, the antenna will receive the backscatter signal from the tag. As discussed in section 2, we treat the current beam direction as AoA. Thus if we directly use formula (5) to calculate the tag’s position, it will introduce a maximum distance error which equals to the radiation radius into the localization result. On the other hand, the width beam lobe will bring in radiation area overlapping problem, which makes the tag being read in multiple directions. In the Fig. 3, *P*_1_, *P*_2_, *P*_3_, *P*_4_ respectively represent the coordinates of the center point of 4 beam directions, and the tag can be read in all the four directions. If we set the reader system to modify its beam on totally *I* directions and record the number of directions that can read the tag as *M*, we can take the quadrangle centroid consists of *M* center coordinates approximation as the actual coordinates of the tag.


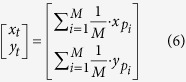


However, we can learn from the Fig. 3 that the actual position of the tag is closer to *P*_3_ while far from *P*_1_. If we can assign another normalized weighting factor for each center point instead of during calculation (6), we can make the tag closer to *P*_3_ and far away from the *P*_1_ in order to further improve the positioning accuracy. The radiation pattern function implies that the near field of the rotation lobe center should have higher radiation intensity. Conversely, the far field contains weaker radiation ability. Since passive tag uses backscatter communication, it means that the tag being close to the radiation center will receive more energy than the one which is far away, thus having higher probability to be more often read. Therefore, in PATL, we count the number of reads in each directions during one inventory as *n*_*i*_, *i* ∈ *M* and provide the normalized weighting factor as


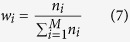


Thus formula (6) is rewritten as


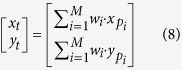


**Figure 4:**
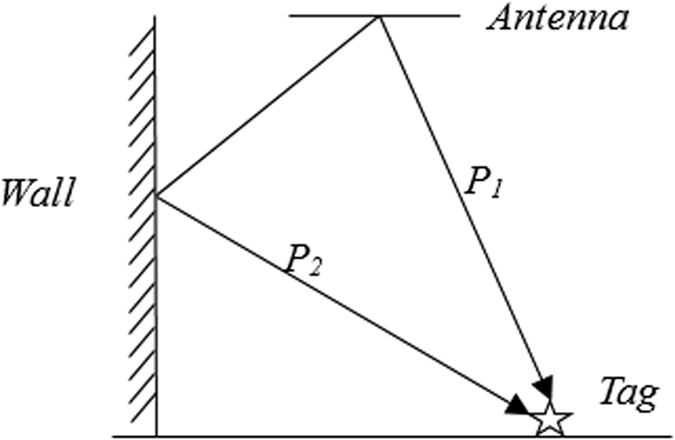
The multiple effect. When there is a reflector (such as the wall) in the environment, the tag will be read in a wrong direction *P_2_*, as well as the correct *P1*.

In addition, the multipath reflection is a problem that must be considered in the practical environment. As shown in the Fig. 4, the tag’s RF signal which is correctly read in the direction of *P*_1_ is also reflected by the wall and is captured by direction *P*_2_. Direction *P*_2_ should not receive the tag signal. Read the tag information from *P*_2_ will add up a large positioning errors when calculating (8). In this case, we want to eliminate the misreading situation.

In brief, we name each adjustable direction as a ‘beam’. We firstly collect the near beams together according to their radiation center positions on the ground and divide them into *K* quadrants. Then after one round inventory by using all of the beams, we sum the number of how many beams in each quadrant that have read the tag. It is denoted as *M*_*k*_ for the *k*^*th*^ quadrant and 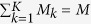. By comparing the beams number *M*_*k*_, we pick up the quadrant *K*_*max*_ with the largest beams number and consider *K*_*max*_ is the quadrant which points to the correct tag position. Otherwise, the reads captured by the beams in the quadrant which is opposite to the *K*_*max*_ quadrant are treated as the results of multipath influence. To this end, before calculating (8), we exclude the multipath influence by comparing the reads number in different direction quadrants. However, due to the thermal noise, the measurement may deviate with a small value. To tolerate the deviation, several adjacent quadrants with high *M*_*k*_, named as candidate quadrants *Q*_*c*_ (the opposite quadrants named as *Q*_*o*_), are selected instead of only using the maximum one. We use three candidate quadrants in this paper. The detailed explanation combining with real experiment measurement data will be described in the follow up section.

So far, we have explained the process of PATL algorithm. It is an AoA based 2D tag localization method. In the next chapter, we will consider RSSI as an auxiliary information to optimize it.

### EPATL

We provide EPATL algorithm on the foundation of PATL to improve the positioning accuracy by optimizing the weighting factor based on the readers’ received signal strength from the tag. According to Friis free-space propagation model^21^, if the reader transmitting power is constant, the tag’s reflection signal strength is only related to the tag-reader distance. Specifically, the received signal strength is proportional to the biquadrate of the reciprocal of the distance. The propagation model can be expressed as formula (9)^22^:


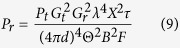


Among them, *P*_*t*_ represents the intensity of the emission power of the reader, *G*_*t*_ represents the transmitter gain of the reader. *G*_*r*_ represents the receiver gain of the tag. *λ* represents the electromagnetic wave length. *d* represents the distance between the transmitter and the receiver. *X* denotes the polarization mismatch. *τ* is the modulation factor. *B* denotes the path-blockage loss. *F* denotes the monostatic fade margin. Θ is the RF tag antenna’s on-object gain penalty, depending on material properties. In reality, *P*_*t*_*, X, F* and Θ are always set to be fixed, *G*_*t*_ and *G*_*r*_ are known from the manufacture. According to ref. 22, the value of *τ* and *B* are 1. Thus the distance *d* between the transmitter and the receiver can be calculated through measuring *P*_*r*_. In wireless communication system, *P_r_* is usually expressed by the received signal strength indicator (RSSI). RSSI is defined as the ratio between the received power and the reference power *P*. Generally, the reference power is set to be 1 mW. The unit of RSSI is dBm.


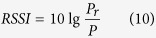


In the indoor environment, formula (9) is approximated to the log path loss model^21^:


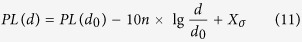


Among them, *PL*(*d*) represents the RSSI of the tag which keeps a distance *d* to the transmitting node. *PL*(*d*_*0*_) represents the RSSI of the reference distance *d*_0_ (generally its value is 1 m). *X*_*σ*_ is used to indicate the measurement error which follows Gaussian random distribution function with zero mean and *σ*^2^ variance. *n* is a radio wave propagation loss coefficient, can be set from 2 to 3. Therefore, in the indoor environment, the relationship between distance *d* and RSSI can be expressed by formula (12):


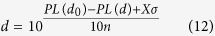


According to formula (12), we know that when the distance between the tag and the reader is closer, the value of RSSI is larger. Thus we can assume that the larger RSSI value is received by the reader, the closer is the tag to the reader. Otherwise, the tag-reader distance gradually increases with the reducing RSSI value. By recognizing this characteristic, we consider to optimize the PATL algorithm based on the RSSI value. As indicated previously, the beam directions generated by phased array antenna has been divided into *K* quadrants and we eliminate the multipath effect by comparing the beams number that can read the tag in each quadrant. After calculating the coordinate values (*x*_*t*_*, y*_*t*_) by PATL algorithm, we attempt to analyze the value of RSSI captured by each beam in the candidate quadrants. The RSSI values are put in order of highest to lowest and the corresponding beam with the maximum RSSI value (or multiple beams with the same value) are selected out, and called candidate beam *B*_*c*_. At the end, we re-calculate the tag’s coordinate value by averaging the center coordinates of *B*_*c*_ and the position result by PATL together as a second weighting process. The specific formulas are as below:


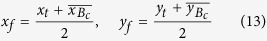


Among them, *x*_*f*_*, y*_*f*_ represent the final calculated coordinate values. *x*_*t*_*, y*_*t*_ represent the PATL algorithm calculated value. 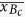, 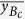 represent the center coordinate of the candidate beam. If there exist multiple candidate beams, it is required to take the mean value of all the center point coordinates of the candidate beams set. We call this second weighting factor method as EPATL which uses RSSI as an auxiliary information.

### Summarize

Since PATL algorithm is a part of EPATL, we summarize the entire EPATL algorithm in Algorithm 1. It shows that this algorithm requires to firstly preprocess the radiation center coordinates (*x_Pi_, y_Pi_*) and separate *I* beams into *K* quadrants as an off-line preparation as soon as the system is established. They constitute the system profile, e.g. *Prof* = {{(*x*_*P1*_*, y*_*P1*_), (*x_P2_, y_P2_*)..}, …, {…, (*x*_*Pi*_*, y*_*Pi*_), …}, …, {…, (*x*_*PI*_*, y*_*PI*_)}}. It is the set of the entire beams center position, each of the secondary bracket means a quadrant. The center coordinate is depended on the height *H* of the antenna. After eliminating the misreading measurements introduced by multipath interference, we use the reads number *n*_*i*_ of the filtered *M* beams to provide the weighting factor and calculate the tag’s 2D coordinate based on PATL. Then we bring in RSSI comparison to give a second weighting optimization as the entire EPATL method.


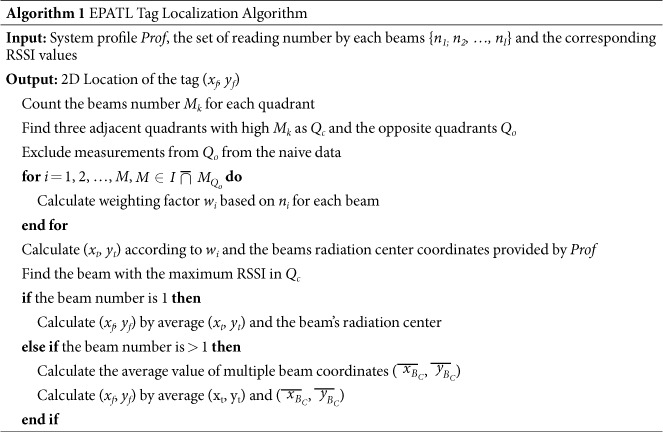


## Experiment Analysis

### Experimental environment

**Figure 5:**
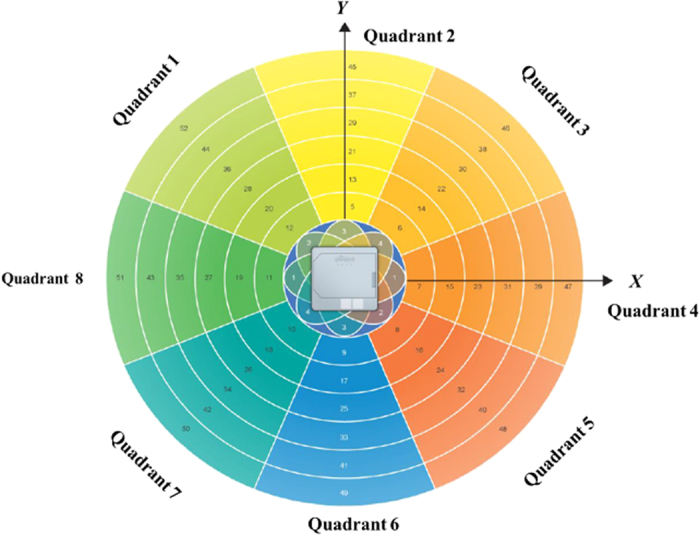
Phased array antenna effect chart^23^.

In this paper, we use Impinj xArray^23^ reader which employed the most advanced technology in the related field for the first time to verify the PATL algorithm. The xArray is a fixed infrastructure UHF RFID reader system that provide wide-area monitoring for real-time identification^23^. It is a reader which integrated with a planar phased array antenna. This planar phased array antenna has 9 antenna elements with a circular distribution. Using digital phase shifter, 52 antenna beam directions can be automatically adjusted. As shown in the Fig. 5, we divide the 52 directions into 8 quadrants and number them by clockwise order. Each quadrant has 7 to 8 beam elements. The reader has the ability to successfully interrogate multiple tags during one inventory and divides their reflection signals based on the unique EPC code^24^. It provides operations based on the EPC LLRP^25^ protocol to support RSSI low level user data.

**Figure 6:**
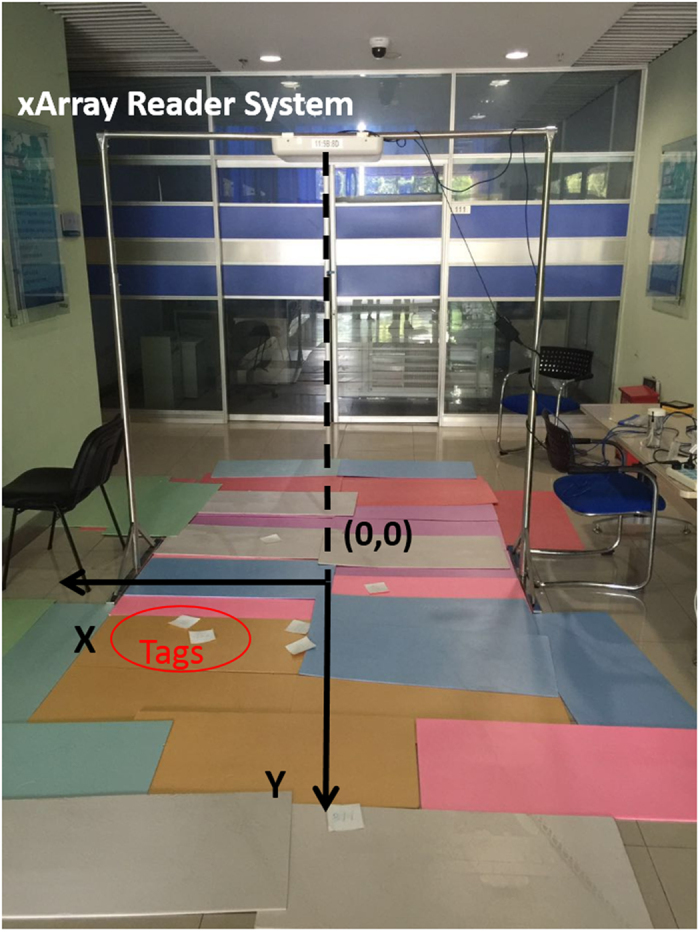
Experimental environment.

We build the experimental environment as shown in the Fig. 6. The xArray reader is hung on 2 meters height. The coordinate system is established based on the projection position of the antenna on the ground. Target tags lie on the ground. According to the internal characteristics of the xArray antenna, the direction angle between the edge beam and the vertical line is 45 degree. Therefore, the radiation area on the ground can be approximated as a circle with a radius of 2 *m*. We calculate formula (5) to give the coordinates of the center position of all the beam directions as shown in the Table 1. As described in section 3, we select three candidate quadrants and the opposite ones are treated as the multipath interference during performing the PATL algorithm. For instance, according to the Fig. 5, if the beam number in quadrant 7, 8, 1 is high, it requires to exclude the reading data in quadrant 3, 4, 5 from the calculation. Similarly, when the reading data in quadrant 1, 2, 3 is extremely high, the data of quadrant 5, 6, 7 are treated as exclusion part. After that, we estimate the position of the tag by using weighting method based on the system profile data in Table 1 and formula (8) and (13).

In the course of our experiment, the type of the target tag is Impinj H47^23^. We use .NET to develop the xArray control program running on the client PC. The reader is set to adjust all the 52 beams one by one during one inventory. The fixed carrier frequency is 920.625 MHz and the transmit power is 30 dBm EIRP. We use Python to count the reading directions and corresponding times, also to execute the PATL and the improved EPATL algorithm. The operating environment is i5 Intel processor at 2.5 GHz and 8G memory Core.

### Experimental result

We divided the experiments into two cases: (1) For the single tag, we used the same tag to repeat the experiments and calculations in a total of 13 different locations. (2) For multi tags situation, we read 8 tags in different locations at once and repeat the reading process again on another location set as a control group to observe the algorithm consistency. We used time-sharing processing of the reading data for different tag via their own unique EPC code to investigate and compare the results. We used both the PATL and the improved EPATL algorithm to detect and calculate the locating results for these two cases, and compare the results.We investigated the performance of the algorithm from two aspects: the average distance error and time consumption. The average distance error (*MD*) between the estimation 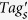 and actual coordinates *Tag_s_* of *S* tags is calculated by:


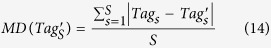


**Figure 7:**
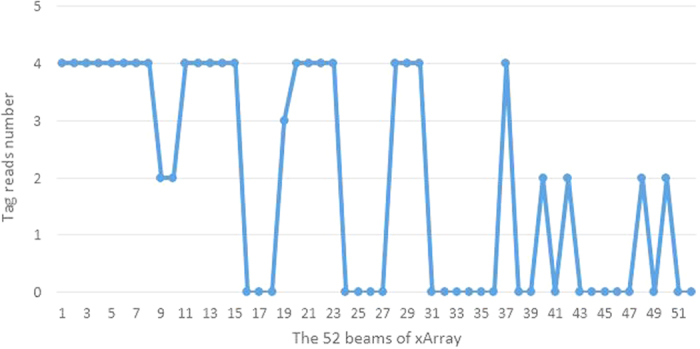
The tag reads number under each beam direction.

**Figure 8:**
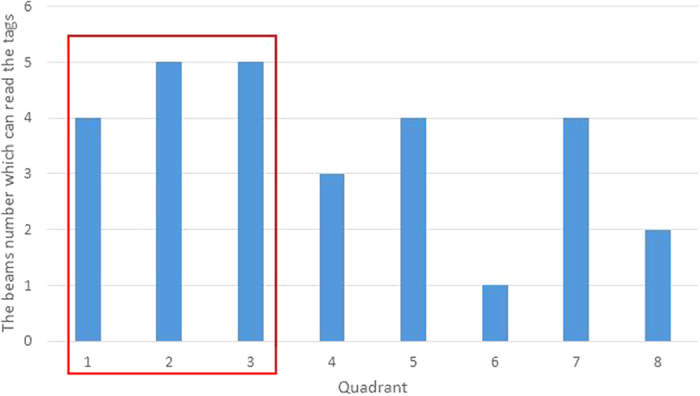
The beam number that can read the tag in each quadrant.

**Figure 9:**
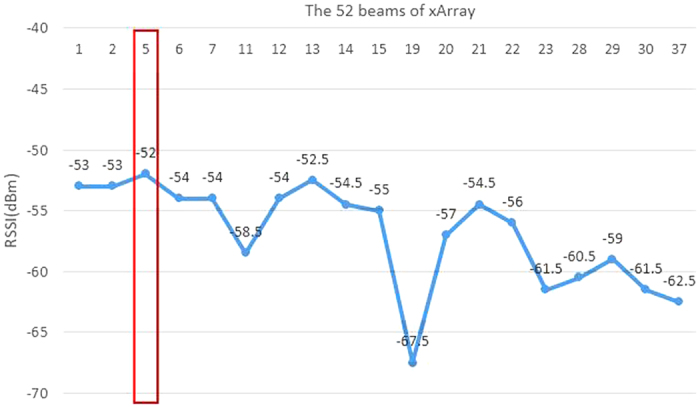
The RSSI values under each beam direction.

Figures 7,8 and 9 give a detailed interpretation of the measurements data when a single tag is at (8, 61). Figure 7 is the tag reads number by the reader in each different beams. From the figure, we can see that only in 1~15, 19~23, 28, 29, 30, 37, 40, 42, 48, 50 beams, the tag is able to be read. The reading number of the rest beams is 0, which indicates that the possibilities of the tag existing in these beams are low. Most of the beams can read the tag 4 times, or at least 2 times during one inventory. However, due to the multipath interference, there are misreading measurements. In order to eliminate the deviation, we separate the beams that can read the tag into 8 quadrants according to Fig. 5 and count the beams number for each quadrant as shown in Fig. 8. From the figure, beams in quadrant 2 and 3 read tag most frequently. Overall, the sum of quadrant 1, 2 and 3 has the highest occupancy rate of reading times, we treat them as the candidate quadrants. Thus the reading measurements in quadrant 5, 6, 7 should be excluded from the original data. By using formula (8), the calculated coordinate result for PATL is (12, 57). Additionally, Fig. 9 shows the RSSI value of each filtered beam. We can learn that the value of RSSI in 5 beam which belongs to quadrant 2 is the biggest, which is −52 dBm. There does not exist the situation that multi beams have the same RSSI value. We directly use formula (13) to give EPATL result as (6, 59). Comparing to PATL, EPATL provides a higher locating precision.

**Figure 10:**
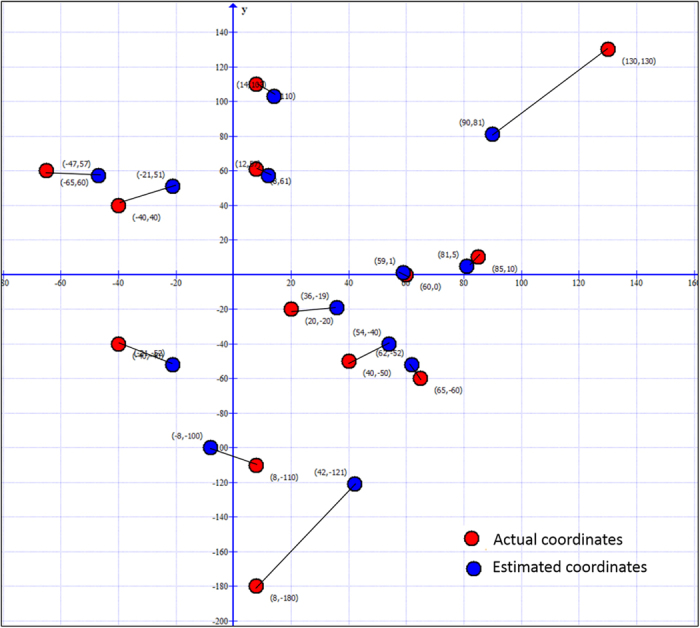
Actual coordinates and estimated coordinates in the single tag experiment of PATL.

We display the specific locating results of PATL for single tag in Fig. 10. The red dots represent the ground truth coordinates and the blue ones represent the estimated coordinates from PATL algorithm. Most of the evaluation results are close to the actual positions, excepting (8, −180) and (130, 130), which have a relatively high locating error. The reason is that the PATL algorithm introduces a trend to pull the estimation result close to the system coordinate origin (0, 0). It is due to the approximate assumption that the tag’s position is close to the centroid of the polygon which is composed by the line segments of beam centers as shown in Fig. 3. Simultaneously, according to the multipath interference, it is also not difficult to give a conclusion that by using PATL algorithm, when the tag is closer to the origin, the error between the estimated and the actual coordinates will be small; while the error will increase with the increasing of tag-reader distance

**Figure 11:**
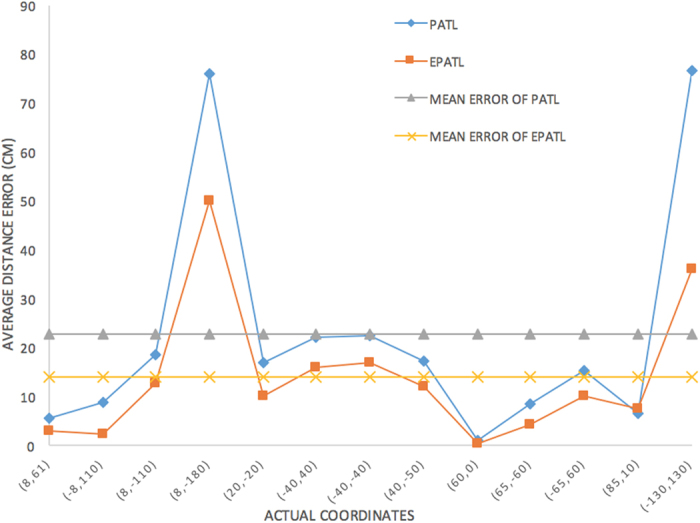
Comparison of single tag’s position calculation error between PATL and EPATL.

In Table 2, we list out the entire results of both PATL and EPATL. The average error of the algorithm PATL is about 21 *cm*, the smallest error is 1 *cm*, 86% point error is less than 20 *cm*. The positioning method compared to ref. 26 based on multiple linear phased array antennas, PATL reduces the locating error by over 15%. Comparing to the optimized fingerprinting method described in ref. 13, PATL improves the accuracy by over 73%. When looking at the EPATL results, we can see that although the locating error of (8, −180) and (130, 130) are still high (within 50 *cm*), EPATL has already increased the accuracy than half comparing to PATL. The mean error of all the 13 positions of EPATL is about 13 *cm*. The calculation error comparison between PATL and EPATL is shown in Fig. 11.

**Figure 12:**
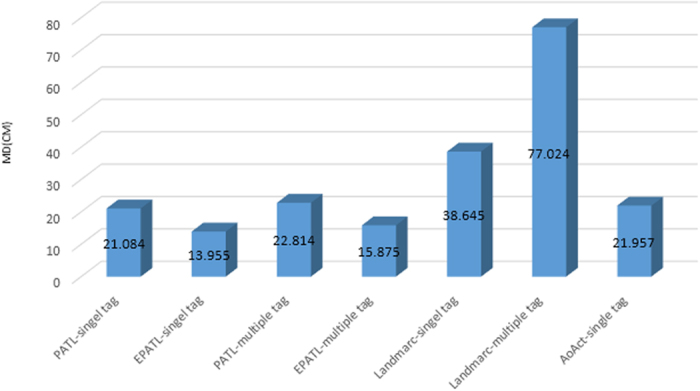
The positioning accuracy vs. different localization schemes.

We have calculated the PATL results of two groups of multiple tags in the Table 3. From the table, we can see that the results between these two groups are similar, which means the PATL algorithm keeps a good consistency. However, because the signal collision phenomenon during the communication process with multi tags, additional interference reduces the positioning accuracy when comparing to the single tag situation. As in the Fig. 12, the mean errors of the PATL and the EPATL for multi tag experiment are around 23 *cm* and 16 *cm*. However, regardless of the single tag or the multi tags cases, EPATL achieves a remarkable accuracy increase when comparing to PATL. At the same time, compared to otherexisting method of LANDMARC^11^ and AoAct^16^, EPATL also has obvious advantages in terms of accuracy. We also investigate the algorithm execution time of PATL and EPATL for both single tag and multi tag situation, excluding the time consumption of tag reading measurements. The execution time of the PATL algorithm is about 3 *ms*. Although EPATL algorithm added the contents of RSSI, the time overhead is not significantly increased with only 3.2 *ms*. It is basically the same as PATL method. Since we use time-sharing method to process multi tags, the execution time will increase with the increasing of the tag number.

## Conclusion

In this paper, we firstly propose a method PATL based on two-dimensional phased array antenna for the RFID system. This method utilizes the feature of the phased array antenna whose radio beam can be automatically adjusted to scan the entire surveillance region.By adding up the tag’s occurrence number in different antenna direction, a weighting factor is used to estimate the 2D position of the tag. The experimental results show that the localization accuracy of the proposed method is 21 *cm*. Additionally, an optimized algorithm EPATL based on RSSI value achieves an accuracy of 13 *cm*. These methods can be applied to the real-time location of multiple targets. The next step is to research moving trajectory tracking for both the single and the multi tags. At the same time, the precision optimization for large scale application is another important research topic.

## Additional Information

**How to cite this article:** Qiu, L. *et al*. PATL: A RFID Tag Localization based on Phased Array Antenna. *Sci. Rep.* **7**, 44183; doi: 10.1038/srep44183 (2017).

**Publisher's note:** Springer Nature remains neutral with regard to jurisdictional claims in published maps and institutional affiliations.

## Figures and Tables

**Table 1:** xArray Phased Array Beam Radiation Center Coordinates.

Quadrant	Beam direction center position coordinates (*m*) format: Beam number (*x, y*)
quadrant 1	1(−0.25, 0.25), 12(−0.44, 0.44), 20(−0.62, 0.62), 28(−0.80, 0.80), 36(−0.97, 0.97), 44(−1.15, 1.15), 52(−1.33, 1.33)
quadrant 2	5(0, 0.625), 13(0, 0.875), 21(0, 1.125), 29(0, 1.375), 37(0, 1.625), 45(0, 1.875)
quadrant 3	2(0.25, 0.25), 6(0.44, 0.44), 14(0.62, 0.62), 22(0.80, 0.80), 30(0.97, 0.97), 38(1.15, 1.15), 46(1.33, 1.33)
quadrant 4	7(0.625, 0), 15(0.875, 0), 23(1.125, 0), 31(1.375, 0), 39(1.625, 0), 47(1.875, 0)
quadrant 5	3(0.25, −0.25), 8(0.44, −0.44), 16(0.62, −0.62), 24(0.80, −0.80), 32(0.97, −0.97), 40(1.15, −1.15), 48(1.33, −1.33)
quadrant 6	9(0, −0.625), 17(0, −0.875), 25(0, −1.125), 33(0, −1.375), 41(0, −1.625), 49(0, −1.875)
quadrant 7	4(−0.25, −0.25), 10(−0.44, −0.44), 18(−0.62, −0.62), 26(−0.80, −0.80), 34(0.97, −0.97), 42(−1.15, −1.15), 50(−1.33, −1.33)
quadrant 8	11(−0.625, 0), 19(−0.875, 0), 27(−1.125, 0), 35(−1.375, 0), 43(−1.625, 0), 51(−1.875, 0)

**Table 2:** Single Tag Estimation Results of PATL and EPATL.

Number	Actual Coordinates *Tag*_*p*_	PATL Coordinates 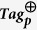	EPATL Coordinate 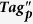	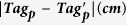	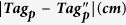
1	(8, 61)	(12, 57)	(6, 59)	5.464	2.828
2	(8, 110)	(14, 103)	(7, 108)	8.946	2.236
3	(8, −110)	(−8, −100)	(−4, −106)	18.658	12.649
4	(8, −180)	(42, −121)	(25, −133)	68.095	49.979
5	(20, −20)	(36, −19)	(30, −22)	16.870	10.198
6	(−40, 40)	(−21, 51)	(41, 56)	21.954	16.031
7	(−40, −40)	(−21, −52)	(−23, −39)	22.472	17.029
8	(40 −50)	(54, −40)	(49, −42)	17.129	12.041
9	(60, 0)	(59, 1)	(60, 0.5)	1.03	0.5
10	(65, −60)	(62, −52)	(62, −57)	8.421	4.242
11	(−65, 60)	(−47, 57)	(−55, 60)	15.297	10
12	(85, 10)	(81, 5)	(84, 2.5)	6.503	7.566
13	(130, 130)	(90, 81)	(106, 103)	63.253	36.124
	MD(cm)			21.084	13.955

**Table 3:** Multi Tag Estimation Results of PATL.

Number	First Group			Second Group		
	Actual Coordinates *Tag*_*P*_	PATL Coordinates 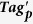	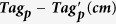	Actual Coordinates *Tag*_*P*_	PATL Coordinates 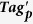	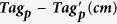
1	(8, 61)	(24, 70)	18.816	(8, −80)	(30, −69)	24.596
2	(8, 110)	(16, 94)	17.888	(60, 0)	(38, 6)	22.803
3	(20, −20)	(42, −15)	22.561	(−20, 20)	(−35, 30)	18.027
4	(40, 40)	(62, 31)	23.769	(40, −40)	(53, −55)	19.849
5	(−40, 50)	(−48, 32)	19.697	(−40, −50)	(−30, −32)	20.591
6	(8, 130)	(14, 102)	28.636	(−60, 0)	(−44, 10)	18.867
7	(65, −60)	(81, −50)	18.867	(−65, 60)	(−57, 50)	12.806
8	(85, 10)	(59, 18)	27.202	(95, 95)	(72, 50)	50.053
MD(cm)	22.1795			23.449		
